# Localisations de la tuberculose chez l’enfant hospitalisé au Complexe hospitalier universitaire pédiatrique de Bangui (République centrafricaine)

**DOI:** 10.48327/mtsi.v6i3.2026.799

**Published:** 2026-09-19

**Authors:** Olivier Brice BOGNING MEJIOZEM, Marie Christine Awa SEPOU YANZA, Laurice NGOZIKA, Junior Sylvere GBELESSO, Abdias NGUERASSEM, Yannick Wilson HENKO KINDING, Jean Chrysostome GODY

**Affiliations:** 1 Complexe hospitalier universitaire pediatrique de Bangui, Republique centrafricaine; 2 Faculte des sciences de la sante, Universite de Bangui, Republique centrafricain; 3 Service des maladies infectieuses et tropicales du Centre hospitalier universitaire de Treichville, Abidjan, Cote d’Ivoire

**Keywords:** Tuberculose extrapulmonaire, Enfant, Bangui, République centrafricaine, Afrique subsaharienne, Extrapulmonary Tuberculosis, Children, Bangui, Central African Republic, Sub-Saharan Africa

## Abstract

**Introduction:**

La tuberculose extrapulmonaire de l’enfant constitue un enjeu majeur de santé publique en raison de sa fréquence non négligeable, de la gravité de ses formes, des difficultés diagnostiques et de son impact sur la morbidité et la mortalité pédiatriques. Cette étude a été initiée avec pour objectif principal de caractériser les aspects de la tuberculose extra-pulmonaire chez l’enfant au Complexe hospitalier universitaire pédiatrique de Bangui (CHUPB).

**Matériels et méthodes:**

Étude rétrospective descriptive menée du 1^er^ janvier 2022 au 30 avril 2024, incluant les enfants de 1 mois à 15 ans hospitalisés au CHUPB, avec un diagnostic de tuberculose extrapulmonaire confirmé dans le dossier médical. Les données ont été saisies sous Microsoft Excel 2016 et analysées avec R-Studio.

**Résultats:**

La fréquence hospitalière était de 1,57 % (287/18 257 enfants hospitalisés). Pour l’étude, seuls 280 dossiers complets ont été analysés. L’âge moyen était de 8,2 ± 3,1 ans, avec un sex-ratio de 1,45. La fièvre prolongée constituait le principal motif de consultation (97,5 %), le délai moyen de consultation était de 1 mois et 6 jours. Le niveau socioéconomique était bas chez 68,5 % des enfants et l’état vaccinal (BCG) incomplet chez 86,7 %. Le contage tuberculeux était noté chez 29 % et l’infection par le VIH chez 23,1 % d’entre eux. La tuberculose était unifocale dans 53,9 % et multifocale dans 46,1 % des cas. Les atteintes neuroméningées représentaient 30,5 %, les localisations ganglionnaires et péritonéales 38,5 %. Le taux de positivité du BAAR était de 66,9 % et celui du test GeneXpert de 33,3 %, avec une résistance à la rifampicine de 12,1 %. Un traitement antituberculeux a été administré à 98,6 % des enfants. Le taux de létalité était de 10,3 % et la durée moyenne d’hospitalisation de 12 jours.

**Conclusion:**

La tuberculose extrapulmonaire chez l’enfant au CHUPB reste fréquente et grave, avec des retards diagnostiques, une faible couverture vaccinale et une mortalité élevée. Pour réduire la morbidité et progresser vers l’Objectif de développement durable 3, il est crucial de renforcer le dépistage et la vaccination, améliorer l’accès aux soins et sensibiliser familles et personnel de santé.

## Abstract (en)

**Introduction:**

Extrapulmonary tuberculosis (TB) in children poses a significant public health challenge due to its high prevalence, severe forms, diagnostic difficulties, and impact on pediatric morbidity and mortality. The primary objective of this study was to characterize extrapulmonary tuberculosis in children at the Bangui Pediatric University Hospital Complex (CHUPB).

**Materials and methods:**

We conducted a retrospective descriptive study from January 1, 2022, to April 30, 2024. We included children aged 1 month to 15 years who were hospitalized at the CHUPB and had a confirmed diagnosis of extrapulmonary tuberculosis in their medical records. The data were entered into Microsoft Excel 2016 and analyzed using R Studio.

**Results:**

The hospitalization rate was 1.57% (287 out of 18,257 hospitalized children). Only 280 complete medical records were analyzed for the study. The mean age was 8.2 ± 3.1 years, with a sex ratio of 1.45. Prolonged fever was the primary reason for seeking medical care (97.5%), and the average delay in seeking care was one month and six days. Low socioeconomic status was observed in 68.5% of the children, and incomplete BCG vaccination status was observed in 86.7% of them. Tuberculosis was present in 29% of cases, and HIV infection was present in 23.1% of them. Tuberculosis was unifocal in 53.9% of cases and multifocal in 46.1%. Neuromeningeal involvement accounted for 30.5% of cases, while lymph node and peritoneal involvement accounted for 38.5%. The smear positivity rate for AFB was 66.9% and that of the GeneXpert test was 33.3%, with a rifampicin resistance rate of 12.1%. Antituberculosis treatment was administered to 98.6% of the children. The case fatality rate was 10.3%, and the mean length of hospitalization was 12 days.

**Conclusion:**

Extrapulmonary tuberculosis remains common and severe among children at the CHUPB, with diagnostic delays, low vaccination coverage, and high mortality rates. In order to reduce morbidity and make progress toward Sustainable Development Goal 3, it is crucial to strengthen screening and vaccination efforts, improve access to care, and raise awareness among families and healthcare workers.

**Figure:**
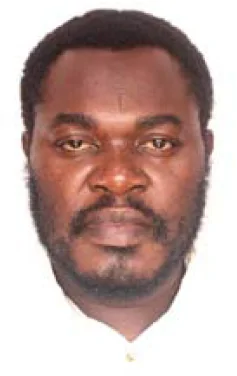

## Introduction

La tuberculose pédiatrique constitue un enjeu majeur de santé publique, en particulier en Afrique, où la charge de morbidité demeure élevée [3]. Selon l’Organisation mondiale de la Santé (OMS), plus d’un million d’enfants sont atteints chaque année dans le monde, représentant environ 10 à 12 % de l’ensemble des cas notifiés de tuberculose. Le continent africain supporte près d’un tiers de cette charge [3,17]. La tuberculose de l’enfant, souvent paucibacillaire, reste difficile à diagnostiquer et à quantifier dans les pays à ressources limitées. Les formes extrapulmonaires, en particulier, posent des difficultés diagnostiques importantes et exposent les enfants à des localisations graves, notamment neuroméningées et miliaires, respon-sables d’une morbidité et d’une mortalité élevées [9,18]. Longtemps considérée comme moins fréquente que la tuberculose pulmonaire, la tuberculose extrapulmonaire représente aujourd’hui 20 à 30% des cas de tuberculose chez l’enfant, dépassant 35 % dans certains pays africains à forte prévalence du VIH, malgré l’objectif de l’OMS d’éliminer la tuberculose d’ici 2035. Cette augmentation est liée à l’épidémie de VIH, à la malnutrition, aux immunodépressions, à l’amélioration des méthodes diagnostiques et aux retards de diagnostic dans les pays à ressources limitées [15,17]. En France, elles représentent environ un quart des cas de tuberculose pédiatrique déclarés [26]. En Afrique, les données restent limitées, et aucune donnée spécifique n’est disponible en République centrafricaine concernant la tuberculose extrapulmonaire en milieu pédiatrique. C’est dans ce contexte que cette étude a été initiée, avec pour objectif principal de déterminer la fréquence hospitalière de la tuberculose extrapulmonaire chez l’enfant au Complexe hospitalier universitaire pédiatrique de Bangui (CHUPB). Elle vise également à décrire les aspects épidémiologiques, diagnostiques, thérapeutiques et le pronostic à court terme de cette affection, afin de contribuer à l’amélioration de sa prise en charge en milieu hospitalier.

## Matériels et méthodes

Il s’agissait d’une étude rétrospective descriptive menée du 1er janvier 2022 au 30 avril 2024 (28 mois) au CHUPB, unique structure spécialisée dans la prise en charge des enfants en détresse vitale en République centrafricaine. La population comprenait tous les enfants âgés de 1 mois à 15 ans hospitalisés dans les unités A et C ainsi qu’en soins intensifs, dont le diagnostic de sortie ou de décès mentionnait une tuberculose extrapulmonaire. Seuls les dossiers médicaux complets ont été inclus. Le diagnostic reposait sur un faisceau d’arguments recueillis dans le dossier médical, notamment anamnestiques (contage tuberculeux avec un cas index proche et chronicité des symptômes) et cliniques, selon la localisation de l’atteinte (méningée, ganglionnaire ou péritonéale). Les données biologiques et radiologiques orientaient également le diagnostic, sans toutefois être spécifiques (hyperprotéi-norachie, hypoglycorachie, pléiocytose, ascite, cloisonnements échographiques et anomalies cérébrales). Lorsqu’ils étaient disponibles, des éléments microbiologiques, notamment la recherche de BAAR ou le test GeneXpert, complétaient les arguments diagnostiques. En raison de la nature paucibacillaire de la tuberculose infantile, certains cas étaient confirmés sur l’ensemble de ces arguments. La bonne réponse au traitement antituberculeux après 15 jours constituait un argument thérapeutique en faveur du diagnostic. En l’absence d’amélioration clinique, l’enfant était réévalué et considéré comme ne répondant pas aux critères diagnostiques de tuberculose, puis exclu de l’étude.

Le traitement suivait le protocole national de prise en charge de la tuberculose, qui prévoit des durées adaptées à la localisation de la maladie : 12 mois pour les formes méningées, 6 mois pour les formes ganglionnaires et entre 9 et 12 mois pour les formes ostéo-articulaires, avec un schéma comportant 2 mois de phase intensive à base d’isoniazide (H), rifampicine (R), pyrazina-mide (Z) et éthambutol (E), suivie d’une phase de continuation de 4 à 10 mois selon la localisation, à base d’isoniazide et rifampicine (HR).

L’échantillonnage était de type exhaustif avec exclusion des dossiers incomplets. Les variables étudiées incluaient les données socio-démogra-phiques, cliniques, paracliniques et thérapeutiques. La collecte des données a débuté après l’approbation du protocole par la Faculté des Sciences de la santé de la République centrafricaine et avec l’accord des responsables des unités concernées. La confidentialité des informations a été strictement respectée, les conflits d’intérêts évités, et les résultats communiqués aux autorités sanitaires du pays. Les données ont été saisies et validées à l’aide de Microsoft Excel 2016, puis analysées avec R-Studio (version 2025.05.1+513). Cette étude a été approuvée par le décanat de la Faculté des Sciences de la santé, Université de Bangui. Le protocole a été évalué et validé par les chefs de départements, chefs d’unités et infirmiers-majors du CHUPB. Le respect de la confidentialité des patients a été strictement assuré.

## Résultats

Au total, 18 257 enfants étaient hospitalisés au CHUPB pendant la période d’étude, dont 287 pour tuberculose extrapulmonaire, soit une fréquence hospitalière de 1,57 %. Parmi eux, 280 enfants répondaient aux critères d’inclusion et ont été retenus pour l’analyse.

Parmi les 280 enfants inclus, 166 étaient des garçons (59,3 %) et 114 des filles (40,7 %), soit un sex-ratio de 1,45. L’âge moyen était de 8,2 ± 3,1 ans, avec des extrêmes de 3 mois à 15 ans. La répartition par tranche d’âge était la suivante : moins de 5 ans, 57 cas (20,4 %), de 5 à 10 ans, 151 cas (53,9 %) et de 10 à 15 ans 72 cas (25,7 %).

Le lieu de résidence était urbain pour 197 enfants (70,3 %) et rural pour 83 (29,7 %). La provenance était le domicile pour 229 enfants (81,8 %), un transfert d’autres formations sanitaires (FOSA) pour 49 enfants (17,5 %) et d’un cabinet médical pour 2 enfants (0,7 %).

Le niveau socio-économique était connu pour 149 enfants. Il était bas chez 102 enfants (68,5 %), moyen chez 46 enfants (30,9 %) et élevé chez un enfant. Parmi les 98 enfants dont l’état vaccinal était connu, le BCG n’était pas à jour chez 87 % et à jour chez 13 %.

La notion de contage tuberculeux était retrouvée chez 51 enfants (29 %). Parmi ces 51 enfants, le cas index était un parent de premier degré pour 41 enfants (80 %) et de deuxième degré pour 10 enfants. La durée moyenne de la symptomatologie était de 1 mois et 6 jours, avec des extrêmes allant de 3 semaines à 21 mois.

Un antécédent médical était retrouvé chez 91 enfants (32,5 %): émaciation sévère (n = 56; 60 %), infection par le VIH (n = 21; 23,1 %), tuberculose pulmonaire (n = 9; 10 %), drépanocytose (n = 3; 3 %) et cardiopathie (n = 2; 2 %).

La triade symptomatique était constituée de fièvre prolongée (97,5 %, n = 273), de toux chronique (75,7 %, n = 212) et d’anorexie associée à l’amaigrissement (57,5 %, n = 161). La répartition des enfants selon la symptomatologie est présentée à la Figure 1.

**Figure 1:**
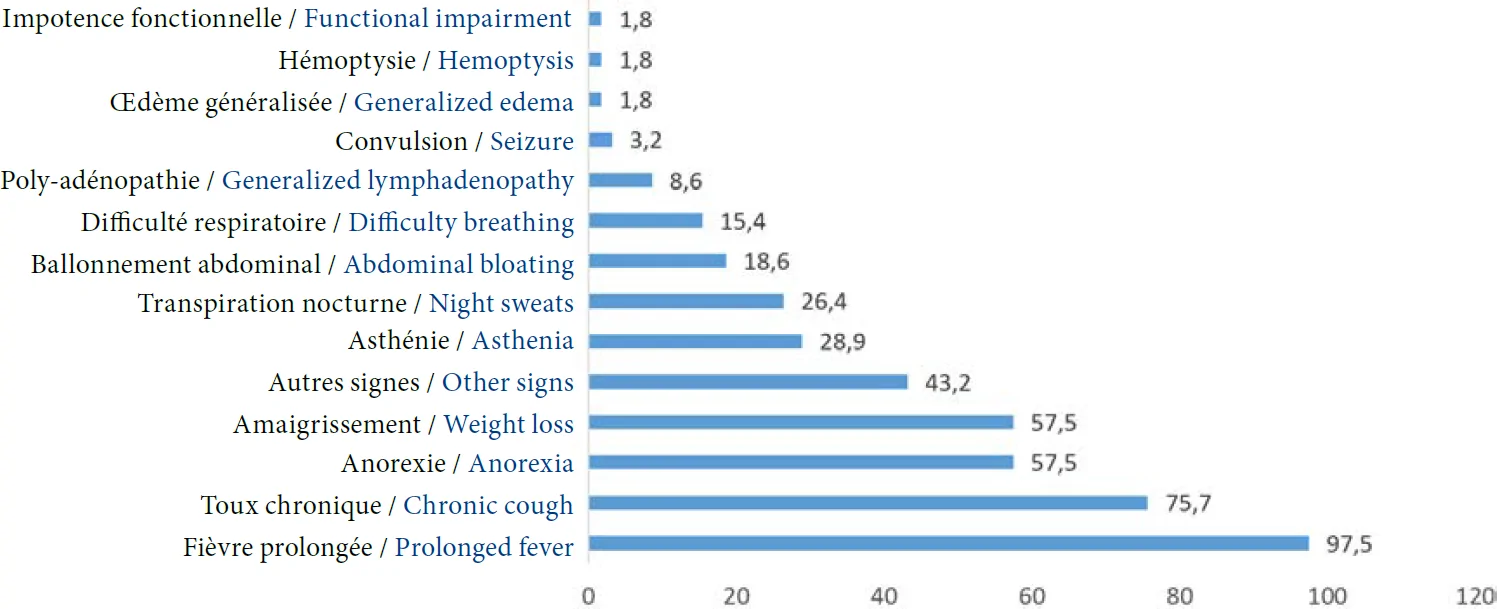
Répartition en pourcentages des enfants en fonction de la symptomatologie. Un même enfant pouvait avoir plusieurs symptômes (N = 280)

Les signes cliniques les plus fréquemment retrouvés étaient la fièvre, la tachycardie (95,7 %) et les adénopathies périphériques (42,9 %). La répartition des enfants en fonction des signes d’examen physique est consignée sur la Figure 2.

**Figure 2:**
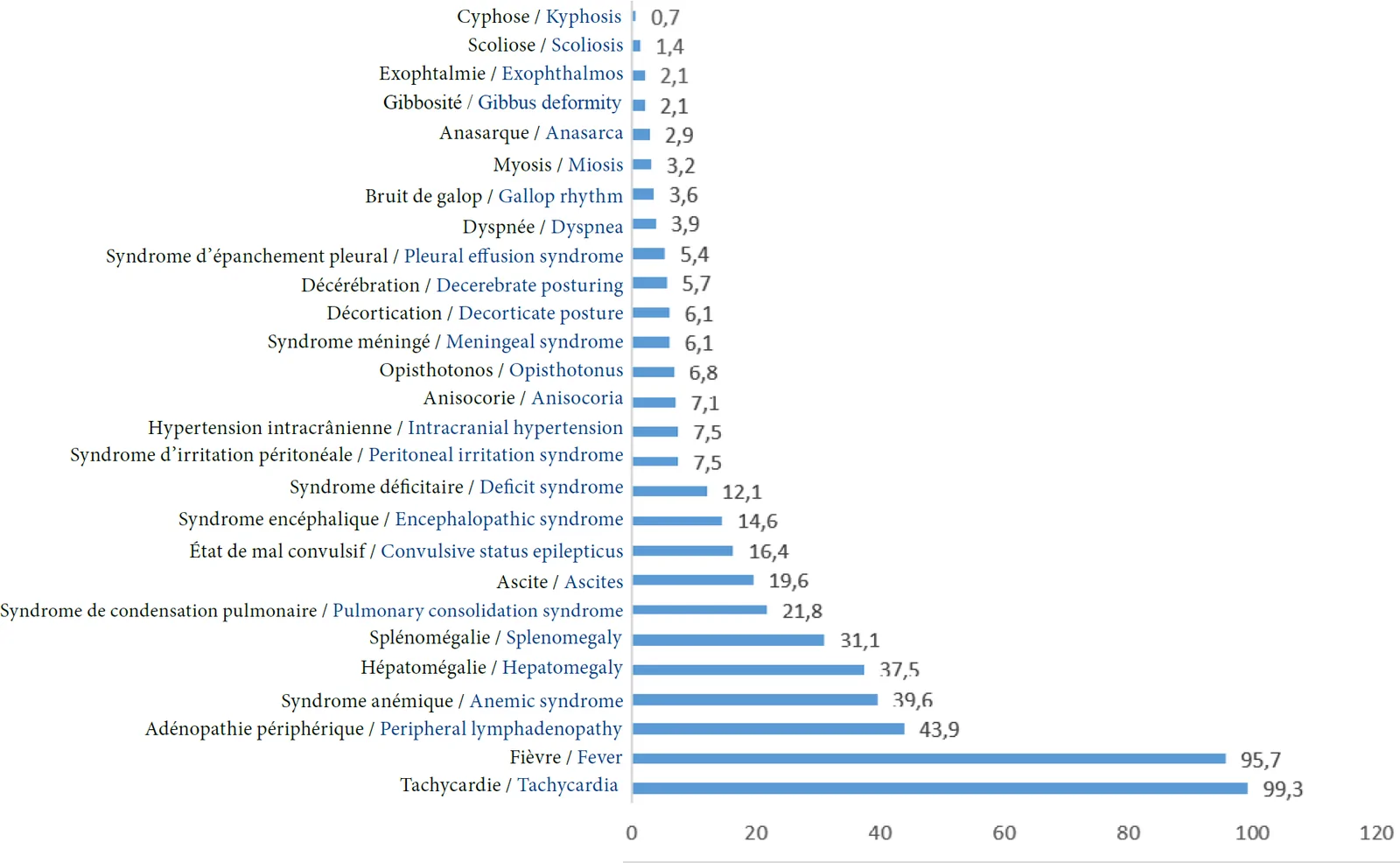
Répartition en pourcentages des enfants en fonction des signes d’examen physique. Un même enfant pouvait avoir plusieurs signes physiques

La tuberculose était unifocale dans 53,9 % (n = 151) et multifocale dans 46,1 % (n = 129). Pour les cas d’atteinte unifocale, les foyers étaient neuromé-ningés (30,5 %, n = 46), ganglionnaires (28,5 %, n = 43), péritonéaux (15,2 %, n = 23), osseux (7,9 %, n = 12), miliaires (7,3 %, n = 11), pleuraux (7,3 %, n = 11), cardiaques (2,6 %, n = 4) et ostéoarticu-laires (0,7 %, n = 1). La localisation ganglionnaire et péritonéale concernait 38,5 % des atteintes bifocales) (Tableau I).

**Tableau I:** Répartition des enfants présentant une atteinte multifocale de la tuberculose selon le type de foyer atteint

Localisation	Effectif	Pourcentage
**Bifocale (n = 117)**		
Ganglionnaire + péritonéale	45	38,5 %
Ganglionnaire + pulmonaire	27	23,1 %
Péritonéale + pulmonaire	26	22,2 %
Pulmonaire + osseuse	5	4,3 %
Ganglionnaire + neuro-méningée	3	2,6 %
Neuro-méningée + pulmonaire	2	1,7 %
Péritonéale + miliaire	2	1,7 %
Péricardiaque+ pulmonaire	2	1,7 %
Péritonéale + cardiaque	2	1,7 %
Ganglionnaire + miliaire	2	1,7 %
Péricardiaque + miliaire	1	0,8 %
**Trifocale (n = 9)**		
Ganglionnaire + pulmonaire + osseuse	2	
Neuro-méningée + pulmonaire+ ganglionnaire	2	
Péritonéale + ganglionnaire+ pulmonaire	1	
Pulmonaire + osseuse + méningée	1	
Pleurale + osseuse + ganglionnaire	1	
Pulmonaire + ganglionnaire + miliaire	1	
Péritonéale + ganglionnaire + pulmonaire	1	
**Quadri-focale (n = 3)**		
Ganglionnaire + pulmonaire + méningée + osseuse	2	
Péricardiaque + pulmonaire + méningée + osseuse	1	

Le taux de positivité de la recherche des bacilles acido-résistants (BAAR) était de 66,9 %(n = 81/121) et le taux de positivité du GeneXpert de 33,3 %(n = 33/99). Parmi les 33 cas positifs au GeneXpert, 29 étaient sensibles à la rifampicine et 4 résistants.

Pour les 16 enfants chez qui une étude du suc ganglionnaire a été effectuée, les résultats étaient: adénite inflammatoire (n = 9), adénite tuberculeuse (n = 6) et adénite aiguë suppurée (n = 1).

Quatrevingt-un enfants (28,9 %) avaient un cliché radiologique et six (2,1 %) avaient réalisé un scanner. La répartition des enfants selon les résultats de la radiographie et du scanner est consignée dans le Tableau II.

**Tableau II:** Résultats des radiographies et scanners

Imagerie	Effectif	Pourcentage
**Radiographies thoraciques (n = 68)**		
Macro-opacité alvéolaire	21	30,9 %
Micronodules miliaires	20	29,4 %
Sans particularité	15	22,1 %
Épanchement pleural	12	17,7 %
**Radiographies rachidiennes (n = 13)**		
Pincement discal	6	
Image en fuseau paravertébral	4	
Pincement discal + image en fuseau paravertébral	1	
Sans particularité	2	
**Scanner cérébral (n = 6)**		
Méningo-encéphalite	2	
Hydrocéphalie tétra-ventriculaire	1	
Leucoencéphalopathie post-hypoxique	1	
Sans particularité	2	

Deux cent soixante-seize enfants (98,6 %) ont reçu un traitement antituberculeux. Quatre enfants (1,4 %) présentaient une résistance à la rifampicine et ont été référés au Service national de prise en charge de la tuberculose résistante pour un traitement de deuxième ligne associant bédaquiline, fluoroquinolone (lévofloxacine ou moxifloxacine) et linézolide. Toutefois, leur évolution n’a pas pu être documentée en raison d’une rupture d’appro-visionnement en médicaments de 2e ligne et de leur perte de vue.

Ainsi, parmi ces 91 enfants, 21 avaient une infection à VIH, soit 23,08 % (21/91). Les antirétrovi-raux étaient suspendus au moment de l’instauration du traitement antituberculeux et repris après deux semaines, en adoptant une approche Traitement observé direct (TOD) pour les deux traitements. Les schémas antirétroviraux reposaient sur des associations à base de dolutégravir (DTG), combiné à deux inhibiteurs nucléosidiques de la transcriptase inverse, notamment la lamivudine (3TC) associée au ténofovir (TDF) ou à l’abacavir (ABC) selon l’âge et le poids. Chez le jeune enfant, l’association ABC + 3TC + DTG était privilégiée.

Le taux de létalité globale était de 10,3 %. La durée moyenne d’hospitalisation était de 12,1 jours, avec des extrêmes de 4 à 45 jours.

Parmi 46 enfants présentant des formes neuro-méningées, 11 (24 %) étaient infectés par le VIH et 14 (30 %) n’avaient pas reçu le vaccin BCG. La mortalité spécifique était de 8 cas (17 %). La durée médiane du traitement antituberculeux était de 12 mois, conformément au protocole national, comprenant 2 mois de phase intensive (HRZE) suivis de 10 mois de phase de continuation (HR) adaptée à la localisation méningée. La durée médiane d’hospitalisation était de 18 jours (extrême de 12 à 26) et 12 enfants (26 %) ont présenté des séquelles neurologiques.

## Discussion

La fréquence de la tuberculose extrapulmonaire était de 1,57 % parmi les enfants hospitalisés. Cette maladie représente environ 20 à 30 % des cas pédiatriques de tuberculose dans différentes séries internationales, avec une large variation selon les régions, allant de 15,9 % en Afrique de l’Ouest [21] à près de 17,8 % en Chine [2], et des proportions plus faibles dans certains centres hospitaliers d’Asie et des Philippines [17], ce qui reflète les difficultés de diagnostic et de notification de cette forme de tuberculose chez l’enfant [21].

L’ampleur globale de la tuberculose pédiatrique reste mal connue, représentant environ 6 % de tous les cas de tuberculose dans le monde, la majorité survenant dans des pays à forte charge de morbidité tuberculeuse [10,21]. Les variations observées entre pays peuvent s’expliquer par la nature paucibacillaire des formes extrapulmonaires et la tendance des cliniciens à privilégier les hypothèses les plus fréquentes selon le contexte épidémiologique local [27,33].

L’âge moyen des enfants hospitalisés pour tuber-culose extrapulmonaire était de 8,2 ans, avec des extrêmes allant de 1 mois à 15 ans. Cette distribution place la majorité des cas dans la tranche d’âge scolaire intermédiaire, ce qui concorde avec d’autres études africaines et internationales. Une étude sénégalaise a rapporté un âge moyen de 7,6 ans [8], tandis qu’une série tunisienne a trouvé une moyenne de 7,5 ans [32]. Des études en Turquie et au Pakistan ont rapporté des médianes de 8,8 ans et des répartitions similaires entre 5 et 14 ans [1,5,11]. Dans les contextes à faible prévalence, comme la République tchèque, l’âge médian est 10 ans, suggérant que l’âge d’apparition varie en fonction du contexte épidémiologique [4]. L’âge est un facteur clé influençant le risque de progression de l’infection tuberculeuse vers la maladie. Les nourrissons et les jeunes enfants (< 5 ans) présentent un risque élevé de développer des formes graves, notamment des formes disséminées ou méningées, en raison de l’immaturité de leur système immunitaire [21]. Dans notre série, bien que la majorité des enfants aient été plus âgés, des nourrissons ont également été touchés, soulignant que la tuberculose extra-pulmonaire peut affecter toutes les tranches d’âge. L’âge moyen observé a des implications cliniques et diagnostiques. Chez les enfants plus âgés, les manifestations cliniques peuvent être plus discrètes ou localisées, entraînant parfois un retard diagnostique [21]. À l’inverse, chez les jeunes enfants, les formes graves sont plus fréquentes et nécessitent une vigilance accrue, un diagnostic rapide et une prise en charge hospitalière adaptée [18,30]. Les retards diagnostiques sont d’autant plus fréquents dans les contextes à ressources limitées, où la présentation clinique non spécifique des formes extrapulmonaires complique la reconnaissance précoce de la maladie [3]. Sur le plan du pronostic, plusieurs études ont montré que les jeunes enfants et les nourrissons présentent une mortalité plus élevée que les enfants plus âgés, notamment en cas de tuberculose extrapulmonaire, en particulier pour les formes méningées et miliaires [9,16]. Dans notre cohorte, malgré un âge moyen relativement élevé, la mortalité restait notable (10,35 %), ce qui suggère que d’autres facteurs, tels que la malnutrition, la coinfection VIH et les retards de prise en charge, interagissent avec l’âge pour déterminer l’issue clinique.

Une prédominance masculine (59 %) a été observée parmi les enfants hospitalisés pour tuberculose extrapulmonaire. Cette tendance a été rapportée dans de nombreuses séries internationales. Au Maroc, Elmghari *et al*. ont décrit 61 % de garçons parmi les enfants atteints de tuberculose extrapulmonaire [6]. À Madagascar, Ramamonjinina *et al*. ont observé 60 % [24] et Kolié *et al*. en Guinée 57 % [13]. En Afrique subsaharienne, d’autres études montrent des proportions similaires, oscillant entre 55 et 62 % [9,29]. La prédominance masculine pourrait s’expliquer par des facteurs biologiques et immunologiques. Les garçons semblent présenter une susceptibilité accrue aux infections durant l’enfance, liée notamment au génotype XY et au rôle limité du chromosome Y dans la réponse immunitaire comparativement au chromosome X, porteur de gènes impliqués dans la défense anti-microbienne [12]. Des facteurs comportementaux ou environnementaux, tels que l’exposition plus précoce aux contacts infectieux dans certaines communautés, peuvent également contribuer [15]. Cependant, certaines séries rapportent une prévalence plus élevée chez les filles, selon les régions et les types d’atteintes extrapulmonaires [17,29]. Cette variabilité pourrait refléter des biais diagnostiques, des différences dans l’accès aux soins ou encore des spécificités locales de transmission familiale et communautaire.

Plus de 86 % des enfants ne présentaient pas un statut vaccinal à jour au BCG. Ce résultat traduit une couverture vaccinale insuffisante dans cette population pédiatrique à haut risque. Cette situation est préoccupante compte tenu du rôle reconnu du vaccin dans la prévention des formes graves de la tuberculose chez l’enfant, notamment les formes méningées et miliaires. Un même constat a été fait en Mauritanie : Salem-Memou *et al*. ont observé une couverture vaccinale au Bacille de Calmette et Guérin de 58,82 % chez les patients atteints de tuberculose extrapulmonaire [25].

Dans notre série, la durée moyenne d’évolution des symptômes avant le diagnostic était de 1 mois et 6 jours, avec des extrêmes allant de 3 semaines à 21 mois, traduisant un délai diagnostique parfois prolongé. Dans le contexte centrafricain, ce retard peut être favorisé par plusieurs facteurs liés au parcours de soins. Une étude menée à Bangui par Longo et *al*. chez des patients atteints de tuberculose pulmonaire bacillifère a montré qu’un délai de consultation supérieur à 30 jours était fréquent (83,1 %) et que le recours aux structures de soins informelles comme premier recours thérapeutique ainsi que l’absence de demande d’examen bactériologique lors de la première consultation étaient associés au retard diagnostique [14]. Ces facteurs, associés aux difficultés d’accès aux structures spécialisées et aux limites du plateau technique diagnostique, pourraient également contribuer au retard de diagnostic des formes tuberculeuses pédiatriques, notamment extrapulmonaires, dont la présentation clinique est souvent moins spécifique.

Plusieurs facteurs structurels et contextuels propres à la République centrafricaine peuvent expliquer cette faible proportion d’enfants correctement vaccinés. D’une part, l’infodémie générée par l’introduction du vaccin contre la Covid-19 dans le pays après la pandémie a contribué à une méfiance accrue envers les programmes de vaccination, limitant ainsi la vaccination systé-matique dès la naissance. D’autre part, certaines croyances au sein des populations rurales selon lesquelles la phytothérapie traditionnelle offrirait une meilleure protection que les vaccins, ainsi que la circulation de rumeurs affirmant que les vaccins seraient destinés à stériliser les populations africaines, peuvent également freiner l’adhésion aux programmes de vaccination. À ces contraintes s’ajoutent l’instabilité sécuritaire et les déplacements fréquents de populations, qui perturbent la continuité des programmes de vaccination et entravent les activités de vaccination de routine et de rattrapage. Les contraintes socio-économiques des ménages, telles que le coût du transport vers les structures de santé ou la faible sensibilisation des parents à l’importance de la vaccination néo-natale, peuvent également contribuer au retard ou à l’absence de vaccination.

Dans ce contexte, un nombre important d’enfants échappe au calendrier vaccinal recommandé dès la naissance. Sur le plan épidémiologique, l’absence de vaccination par le BCG est associée à un risque accru de développer des formes extrapulmonaires sévères chez l’enfant. Plusieurs travaux ont montré que, bien que son efficacité contre la tuberculose pulmonaire soit variable, le BCG réduit signifi-cativement la morbidité et la mortalité liées aux formes disséminées de la tuberculose durant la petite enfance [3,7].

Dans notre série, le taux de positivité du GeneXpert était inférieur à celui de la recherche des BAAR. Cette différence ne traduit pas néces-sairement une moindre performance diagnostique du GeneXpert, mais s’explique principalement par des contraintes organisationnelles et techniques propres à notre contexte. En effet, le GeneXpert n’était pas disponible de façon permanente au Centre Hospitalier Universitaire Pédiatrique de Bangui. Les examens réalisés ont été obtenus grâce à une collaboration avec l’Institut Pasteur de Bangui, qui a assuré gratuitement leur réalisation après négociation au cas par cas. À l’inverse, la recherche des BAAR est réalisée gratuitement au Laboratoire National de Biologie Clinique et de Santé Publique de Bangui, ce qui la rend plus accessible et explique son utilisation plus fréquente en pratique courante ainsi que le nombre plus élevé de prélèvements analysés. Les deux examens n’ont donc pas été réalisés chez une population identique, ce qui ne permet pas de comparer directement leurs performances diagnostiques. Par ailleurs, plusieurs études ont montré que le GeneXpert présente une sensibilité supérieure à celle de la microscopie des BAAR, en particulier dans les formes paucibacillaires fréquentes chez l’enfant, tout en permettant la détection rapide de la résistance à la rifampicine, ce qui en fait l’examen moléculaire de référence recommandé par l’Organisation mondiale de la Santé pour le diagnostic de la tuberculose pédiatrique [19,22]. Dans notre série, 54 % des enfants présentaient une atteinte unifocale, dominée par les localisations cérébro-méningée (30 %), ganglionnaire (28 %) et péritonéale (15 %). Cette répartition est conforme aux données de la littérature en particulier dans les pays à forte endémie tuberculeuse [3,9,24]. La prédominance des atteintes cérébro-méningées souligne la gravité de la tuberculose extrapulmonaire pédiatrique et l’importance d’un diagnostic précoce, ces formes étant associées à une morbidité et une mortalité élevées en l’absence de prise en charge rapide [31].

Le taux de létalité liée à la tuberculose extrapul-monaire était de 10 %. Ce taux élevé s’explique en grande partie par la fréquence des formes graves, notamment les atteintes cérébro-ménin-gées, ainsi que par les retards diagnostiques et thérapeutiques observés en contexte de ressources limitées [1,4,11,24]. Des taux de mortalité comparables ont été rapportés dans plusieurs études africaines et asiatiques, variant entre 8 % et 20 %, particulièrement en cas de coinfection par le virus de l’immunodéficience humaine ou de malnutrition sévère [3,28]. À l’inverse, les pays disposant d’un diagnostic précoce et d’un plateau technique adéquat rapportent des mortalités plus faibles, généralement inférieures à 5 %, soulignant l’impact déterminant de la précocité de la prise en charge [23,24].

L’évolution des différentes localisations tuber-culeuses était variable selon le site d’atteinte. Les formes ganglionnaires, pleurales et péritonéales ont présenté une évolution clinique favorable sous traitement antituberculeux, avec une régression progressive des signes cliniques au cours du suivi. Les formes ostéo-articulaires ont nécessité, en complément du traitement antituberculeux, une immobilisation prolongée et une surveillance spécialisée selon l’atteinte concernée. Les atteintes péricardiques ont été prises en charge par un traitement antituberculeux associé à une corticothérapie, avec une évolution clinique favorable sous surveillance. Les formes neuro-méningées ont également bénéficié d’une corticothérapie adjuvante au traitement antituberculeux; elles représentaient les localisations associées au pronostic le plus défavorable, avec une mortalité de 17 % et la survenue de séquelles neurologiques chez 26 % des survivants. Ces résultats concordent avec les recommandations de l’Organisation mondiale de la Santé, qui considèrent la tuberculose neuro-méningée comme une forme grave nécessitant une prise en charge spécifique, associant un traitement antituberculeux adapté et une corticothérapie adju-vante, en raison du risque élevé de complications neurologiques, de séquelles et de mortalité [20]. Notre approche rétrospective et monocentrique li-mite la généralisation des résultats à l’ensemble de la population pédiatrique centrafricaine. De plus, l’accès inégal aux tests diagnostiques sensibles peut avoir sousestimé la proportion réelle de tuberculose extrapulmonaire dans notre cohorte. Le CHUPB est le principal centre de référence pédiatrique national, drainant la majorité des enfants de Bangui dans un état critique, conférant une représentativité partielle des cas graves. Les données immunovirologiques détaillées (CD4, charge virale, observance du traitement anti-rétroviral) n’étaient pas disponibles. De même, les dossiers médicaux ne permettaient pas de documenter les causes de l’échec du traitement antituberculeux chez les enfants ayant un anté-cédent de tuberculose pulmonaire, notamment l’observance thérapeutique, les interruptions de traitement ou une éventuelle résistance aux antituberculeux.

## Conclusion

La tuberculose extrapulmonaire chez l’enfant au CHUPB reste fréquente et grave, avec des retards diagnostiques, une couverture vaccinale insuffisante et une mortalité élevée. Pour sauver des vies et avancer vers l’Objectif de développement durable 3, le gouvernement centrafricain doit renforcer le dépistage précoce de la tuberculose. Il est également nécessaire de garantir la vaccination complète par le BCG et d’améliorer l’accès aux soins pédiatriques spécialisés ainsi qu’aux traitements antituberculeux. Enfin, la sensibilisation des familles et du personnel de santé reste un volet crucial de la lutte contre cette maladie. Ces actions sont indispensables pour réduire la morbidité et la mortalité liées à la tuberculose pédiatrique.

## Financement

Aucun financement spécifique n’a été reçu pour la réalisation de cette étude. Les auteurs déclarent que la réalisation de ce manuscrit n’a bénéficié d’aucune subvention ni soutien institutionnel ou commercial.

## Contributions des auteurs et autrices

BOGNING MEJIOZEM Olivier Brice : conceptualisation, méthodologie, conservation des données, rédaction, révision et édition, rédaction de la version originale, administration de l’étude.

SÉPOU Yanza Marie Christine Awa: supervision, révision et édition. NGOZIKA Laurice: conceptualisation, investigation, conservation des données, administration de l’étude. GBELESSO Junior Sylvère : investigation, conservation des données et rédaction. NGUERASSEM Abdias : analyse des données et relecture. HENKO KINDING Yannick Wilson : collecte des données et lecture critiques. GODY Jean Chrysostome: supervision, validation de l’étude

## Déclaration de liens d’intérêt

Aucun lien d’intérêt n’a été déclaré.
