# Supplementary material for: Locations of tuberculosis in children hospitalized at the Bangui Pediatric University Hospital Complex (Central African Republic)
Source: Med Trop Sante Int. 2026 Sep 19;6(3):mtsi.v6i3.2026.799. [Article in French] doi: 10.48327/mtsi.v6i3.2026.799 (PMC13697914; doi:10.48327/mtsi.v6i3.2026.799)
Supplement: Related texts

# Research article: Locations of tuberculosis in children hospitalized at the Bangui Pediatric University Hospital Complex (Central African Republic)

## Introduction

Pediatric tuberculosis is a significant public health concern, especially in Africa, where the disease burden remains high [3]. According to the World Health Organization (WHO), more than one million children are affected by the disease world-wide each year, accounting for 10 to 12 percent of all reported cases of tuberculosis. The African continent bears nearly one-third of this burden [3,17]. Low-bacillary tuberculosis in children is often difficult to diagnose and quantify in countries with limited resources. Extrapulmonary forms, in particular, pose significant diagnostic challenges and expose children to severe localizations, notably neuromeningeal and miliary, which are responsible for high morbidity and mortality [9,18]. Although long considered less common than pulmonary tuberculosis, extrapulmonary tuberculosis now accounts for 20 to 30 percent of tuberculosis cases in children and exceeds 35 percent in some African countries with high HIV prevalence. This exceeds the WHO’s goal of eliminating tuberculosis by 2035. This increase is linked to the HIV epidemic, malnutrition, immunosuppression, improved diagnostic methods, and delays in diagnosis in resource-limited countries [15,17]. In France, extrapulmonary tuberculosis accounts for approximately one-quarter of reported pediatric tuberculosis cases [26]. In Africa, data are limited, and no specific data are available in the Central African Republic regarding extrapulmonary tuberculosis in children. In this context, this study was initiated to determine the hospital prevalence of extrapulmonary tuberculosis in children at the Bangui Pediatric University Hospital Complex (CHUPB). The study also aims to describe the epidemiological, diagnostic, and therapeutic aspects of this condition, as well as its short-term prognosis, in order to improve its management in a hospital setting.

## Materials and methods

This retrospective descriptive study was conducted from January 1, 2022, to April 30, 2024 (i.e. 28 month), at the CHUPB. The CHUPB is the only facility in the Central African Republic that specializes in the care of critically ill children. The study population included all children between the ages of 1 month and 15 years who were hospitalized in Units A and C, as well as in the intensive care unit, and who were discharged or died with a diagnosis of extrapulmonary tuberculosis. Only complete medical records were included. The diagnosis was based on evidence from the medical records, including anamnesis (history of tubercu-losis exposure to a close index case and chronicity of symptoms), clinical signs depending on the location (e.g., meningeal, lymph node, and peritoneal), and laboratory and radiological findings that support the diagnosis but none are specific to it (e.g., hyperproteinorachia, hypoglycorachia, pleocytosis, ascites, ultrasound septations, and cerebral abnormalities). When available, the diagnosis was also based on microbiological findings (e.g., AFB or GeneXpert). Due to the paucibacillary nature of childhood tuberculosis, some cases were confirmed based on all these criteria. A good response to antituberculosis treatment after 15 days supported the diagnosis. However, in the absence of clinical improvement, the child was reevaluated and found not to meet the diagnostic criteria for tuberculosis. Consequently, the child was excluded from the study.

Treatment followed the national tuberculosis management protocol, which specifies treatment durations tailored to the site of the disease: Twelve months for meningeal forms, six months for lymph node forms, and nine to 12 months for osteoarticular forms. The regimen consists of an intensive phase of two months based on isoniazid (H), rifampicin (R), pyrazinamide (Z), and ethambutol (E). This is followed by a continuation phase of four to 10 months, depending on the site of infection and consisting of isoniazid and rifampicin (HR).

The sampling was exhaustive, excluding incomplete records. The variables studied included sociodemographic, clinical, paraclinical, and therapeutic data. Data collection began after the protocol was approved by the Faculty of Health Sciences of the Central African Republic and the relevant unit heads gave their consent. Confidentiality was strictly maintained, conflicts of interest were avoided, and the results were communicated to the country’s health authorities. The data were entered and validated using Microsoft Excel 2016 and analyzed using R Studio (version 2025.05.1+513).

The Dean’s Office of the Faculty of Health Sciences at the University of Bangui approved this study. The protocol was reviewed and approved by department heads, unit heads, and head nurses at the CHUPB. Patient confidentiality was strictly maintained.

## Results

A total of 18,257 children were hospitalized at the CHUPB during the study period, including 287 children with extrapulmonary tuberculosis, representing a hospitalization rate of 1.57%. Of these children, 280 met the inclusion criteria and were included in the analysis.

Of those, 166 were boys (59.3%) and 114 were girls (40.7%), yielding a sex ratio of 1.45. The mean age was 8.2 ± 3.1 years, ranging from three months to 15 years. The distribution by age group was as follows: under 5 years, 57 cases (20.4%), 5 to 10 years, 151 cases (53.9%), and 10 to 15 years 72 cases (25.7%).

Of the children, 197 (70.3%) lived in urban areas and 83 (29.7%) lived in rural areas. The point of origin was the child’s home for 229 children (81.8%), a transfer from other health facilities (FOSA) for 49 children (17.5%), and a doctor’s office for two children (0.7%).

Socioeconomic status was available for 149 children. It was low for 102 children (68.5%), moderate for 46 children (30.9%), and high for one child. Of the 98 children for whom vaccination status was known, 87% were not up to date on their BCG vaccinations, while 13% were up to date.

Evidence of tuberculosis transmission was found in 51 children (29%). Of these 51 children, the index case was a first-degree relative for 41 children (80%) and a second-degree relative for 10 children. The average symptom duration was one month and six days, ranging from three weeks to 21 months.

A medical history was identified in 91 children (32%): severe wasting (n = 56; 60%), HIV infection (n = 21; 23.1%), pulmonary tuberculosis (n = 9; 10%), sickle cell disease (n = 3; 3%), and heart disease (n = 2; 2%).

The symptomatic triad consisted of prolonged fever (97.5%, n = 273), chronic cough (75.7%, n = 212), and anorexia with weight loss (57.5%, n = 161). Figure 1 shows the distribution of children according to their symptoms.

**Figure 1:** Percentage distribution of children based on symptoms. A single child could have multiple symptoms (N = 280)

The most frequently observed clinical signs were fever (95.7%) and tachycardia (42.9%), as well as peripheral lymphadenopathy. Figure 2 shows the distribution of children according to physical examination findings.

**Figure 2:** Percentage distribution of children based on physical examination findings. A single child could exhibit multiple physical signs

Tuberculosis was unifocal in 53.9% (n = 151) of cases and multifocal in 46.1% (n = 129) of cases. In cases of unifocal involvement, the foci were meningeal (30.5%, n = 46), lymph node (28.5%, n = 43), peritoneal (15.2%, n = 23), bone (7.9%, n = 12), miliary (7.3%, n = 11), pleural (7.3%, n = 11), cardiac (2.6%, n = 4), and osteoarticular (0.7%, n = 1). Lymph node and peritoneal involvement accounted for 38.5% of bifocal cases (Table I). The positivity rate for acid-fast bacilli (AFB) testing was 66.9%, while the positivity rate for the GeneXpert test was 33.3% (n = 33/99). Of the 33 GeneXpert-positive cases, 29 were susceptible to rifampin, while four were resistant.

**Table 1:** Distribution of children with multifocal tuberculosis by type of affected site

Location	No of children	Percentage
**Bifocal (n = 117)**		
Lymph node + peritoneal	45	38,5 %
Lymph node + pulmonary	27	23,1 %
Peritoneal + pulmonary	26	22,2 %
Pulmonary + bone	5	4,3 %
Neurological/meningeal + lymph node	3	2,6 %
Neurological/meningeal + pulmonary	2	1,7 %
Peritoneal + miliary	2	1,7 %
Pericardial + pulmonary	2	1,7 %
Peritoneal + cardiac	2	1,7 %
Lymph node + miliary	2	1,7 %
Pericardial + miliary	1	0,8 %
**Trifocal (n = 9)**		
Lymph node + pulmonary + bone	2	
Neurological/meningeal + pulmonary + lymph node	2	
Peritoneal + lymph node + pulmonary	1	
Pulmonary + bone + meningeal	1	
Pleural + bone + lymph node	1	
Pulmonary + lymph node + miliary	1	
Peritoneal + lymph node + pulmonary	1	
**Quadrifocal (n = 3)**		
Lymph node + pulmonary + meningeal + bone	2	
Pericardial + pulmonary + meningeal + bone	1	

Of the 16 children who underwent lymph node fluid analysis, nine had inflammatory adenitis, six had tuberculous adenitis, and one had acute suppurative adenitis.

Eighty-one children (28.9%) had chest X-rays, and six (2.1%) had CT scans. Table II shows the distribution of children according to the results of the chest X-ray and CT scan.

**Table II:** X-ray and CT Scan Results

Imaging	No of children	Percentage
**Chest X-rays (n = 68)**		
Alveolar opacity	21	30,9 %
Miliary micronodules	20	29,4 %
No abnormal findings	15	22,1 %
Pleural effusion	12	17,7 %
**Spine X-rays (n = 13)**		
Disc space narrowing	6	
Paravertebral fusiform opacity	4	
Disc space narrowing + paravertebral fusiform opacity	1	
No abnormal findings	2	
**Brain CT scan (n = 6)**		
Meningoencephalitis	2	
Four-ventricle hydrocephalus	1	
Post-hypoxic leukoencephalopathy	1	
No abnormal findings	2	

Two hundred seventy-six children (98.6%) received antituberculosis treatment. Four children (1.4%) were rifampin-resistant and were referred to the National Service for the Management of Drug-Resistant Tuberculosis for second-line treatment combining bedaquiline, a fluoroquinolone (levo-floxacin or moxifloxacin), and linezolid. However, their clinical course could not be documented due to a shortage of second-line medications and loss of follow-up.

Thus, among these 91 children, 21 had HIV, or 23.08% (21/91). For the 21 children (23.1%) infected with HIV, antiretroviral therapy was suspended at the start of antituberculosis treatment and re-sumed after two weeks using a directly observed therapy (DOT) approach for both regimens. The antiretroviral regimens were based on combinations containing dolutegravir (DTG) combined with two nucleoside reverse transcriptase inhibitors: lamivudine (3TC) combined with tenofovir (TDF) or abacavir (ABC), depending on the child’s age and weight. For young children, the ABC + 3TC + DTG regimen was preferred.

The overall casefatality rate was 10.3%. The mean length of hospital stay was 12.1 days, ranging from four to 45 days.

Of the 46 children with meningeal tuberculosis, 11 (24%) were HIV-positive, and 14 (30%) had not received the BCG vaccine. The case-specific mortality rate was 17%. The median duration of antituberculosis treatment was 12 months, in accordance with the national protocol. This treatment consisted of a two-month intensive phase (HRZE), followed by a 10-month continuation phase (HR), adapted to the meningeal location. The median length of hospitalization was 18 days (range 12 to 26), and 12 children (26%) developed neurological sequelae.

## Discussion

The prevalence of extrapulmonary tuberculosis was 1.57% among hospitalized children. This disease accounts for 20% to 30% of pediatric tuberculosis cases in various international studies, with significant regional variation ranging from 15.9% in West Africa [21] to nearly 18% in China [2], and lower rates in some Asian hospitals and the Philippines [17], reflecting the challenges of diagnosing and reporting this form of tuberculosis in children [21].

The global burden of pediatric tuberculosis re-mains poorly understood. It accounts for approximately 6% of all tuberculosis cases worldwide, with most occurring in countries with a high tuberculosis burden [10,18]. Variations between countries can be explained by the paucibacillary nature of extrapulmonary forms and clinicians’ tendency to favor common hypotheses based on local epidemiological contexts [27,33].

The mean age of children hospitalized for extrapulmonary tuberculosis was 8.2 years, with ages ranging from one month to 15 years. This distribution places most cases in the middle school age range, consistent with other African and international studies. A Senegalese study reported a mean age of 7.6 years [8], and a Tunisian study found a mean age of 7.5 years [32]. Studies in Turkey and Pakistan reported medians of 8.8 years and similar distributions between 5 and 14 years [1,5,11]. In low-prevalence settings such as the Czech Republic, the median age is 10 years, suggesting that the age of onset varies by epide-miological context [4]. Age is a key factor in the progression from tuberculosis infection to disease.

Infants and young children (under 5 years old) are at high risk of developing severe forms of the disease, particularly disseminated or meningeal forms, due to the immaturity of their immune systems [21]. In our series, although most of the children were older, infants were also affected, demonstrating that extrapulmonary tuberculosis can affect all age groups. The observed mean age has clinical and diagnostic implications. In older children, clinical manifestations may be more subtle or localized, sometimes leading to a delay in diagnosis [21]. Conversely, severe forms are more common in young children and require in-creased vigilance, rapid diagnosis, and appropriate hospital care [18,30]. Diagnostic delays are more common in settings with limited resources, where the non-specific clinical presentation of extrapulmonary forms complicates the early recognition of the disease [3]. Several studies have shown that young children and infants have higher mortality rates than older children, particularly in cases of extrapulmonary tuberculosis, especially the meningeal and miliary forms [9,16]. Despite the relatively high mean age in our cohort, mortality remained significant at 10.35%, suggesting that factors such as malnutrition, HIV coinfection, and delays in treatment interact with age to determine clinical outcome.

Among children hospitalized for extrapulmonary tuberculosis, a predominance of male patients (59%) was observed. This trend has been reported in numerous international studies. In Morocco, Elmghari *et al.* found that 61% of children with extrapulmonary tuberculosis were male [6]. In Madagascar, Ramamonjinina *et al.* observed 60% [24], and in Guinea, Kolié *et al.* observed 57% [13]. Other studies in sub-Saharan Africa show similar proportions ranging from 55% to 62% [9,29]. The predominance of males could be explained by biological and immunological factors. Boys appear to be more susceptible to infections during childhood due to the XY genotype and the Y chromosome’s limited role in the immune response compared to the X chromosome, which carries genes involved in antimicrobial defense [12]. Behavioral or environmental factors, such as earlier exposure to infectious agents in certain communities, may also contribute to this trend [15]. However, some studies report a higher prevalence among girls depending on the region and type of extrapulmonary involvement [17,29]. This variability may reflect diagnostic biases, differences in access to care, or local patterns of family and community transmission.

More than 86% of the children did not have an up-to-date BCG vaccination status. This indicates insufficient vaccination coverage in this high-risk pediatric population. This is concerning given the recognized role of the vaccine in preventing severe forms of tuberculosis in children, particularly the meningeal and miliary forms. A similar finding was reported in Mauritania: Salem-Memou *et al.* observed a BCG vaccination coverage rate of 58.82% among patients with extrapulmonary tuberculosis (EPTB) [25].

In our series, the average duration of symptoms before diagnosis was one month and six days, ranging from three weeks to 21 months. This indicates that the time to diagnosis was sometimes prolonged. In the Central African context, this delay may be exacerbated by factors related to the care pathway. A study conducted in Bangui by Longo *et al.* among patients with bacilliferous pulmonary tuberculosis revealed that delays exceeding 30 days in seeking medical care were common (83.1%) and that using informal healthcare facilities as the initial point of care and failing to request a bacteriological test during the initial consultation were associated with diagnostic delays [14]. These factors, combined with difficulties accessing specialized facilities and limited diagnostic capabilities, could contribute to delayed diagnoses of pediatric tuberculosis, particularly extrapulmonary forms, whose clinical presentations are often less specific.

Several structural and contextual factors specific to the Central African Republic may explain the low proportion of properly vaccinated children. The infodemic generated by the introduction of the SARS-CoV-2 vaccine following the pandemic has contributed to increased mistrust of vaccination programs, thereby limiting routine vaccinations from birth. On the other hand, certain beliefs among rural populations—name-ly, that traditional herbal medicine offers better protection than vaccines—as well as rumors claiming that vaccines are intended to sterilize African populations may hinder adherence to vaccination programs. Added to these constraints are security instability and frequent population displacement, which disrupt the continuity of vaccination programs and hinder routine and catch-up vaccination activities. Socioeconomic constraints faced by households, such as the cost of transportation to health facilities or parents’ lack of awareness about the importance of neonatal vaccination, can contribute to delays or failure to vaccinate.

In this context, a significant number of children do not receive the recommended immunizations from birth. From an epidemiological perspective, not receiving the BCG vaccine is associated with an increased risk of developing severe extrapulmonary forms of tuberculosis in children. Although its efficacy against pulmonary tuber-culosis is variable, several studies have shown that BCG significantly reduces morbidity and mortality associated with disseminated forms of tuberculosis during early childhood [3,7].

In our study, the GeneXpert positivity rate was lower than the rate of AFB testing. This difference does not necessarily reflect the GeneXpert test’s inferior diagnostic performance, but is primarily due to organizational and technical constraints specific to our setting. The GeneXpert test was not permanently available at the Bangui Pediatric University Hospital. Tests were performed in collaboration with the Pasteur Institute in Bangui, which provided them free of charge following case-by-case negotiations. In contrast, AFB testing is free at the National Laboratory of Clinical Biology and Public Health in Bangui, making it more accessible and explaining its more frequent use in routine practice, as well as the higher number of specimens analyzed. Therefore, the two tests were not performed on the same population, precluding a direct comparison of their diagnostic performance. Furthermore, several studies have shown that the GeneXpert test is more sensitive than AFB microscopy, particularly for paucibacillary forms, which are common in children. The GeneXpert test can also rapidly detect rifampicin resistance, making it the gold-standard molecular test recommended by the World Health Organization for diagnosing pediatric tuberculosis [19,22].

In our study, 54% of children had unifocal infections, primarily affecting the central nervous system (30%), lymph nodes (28%), and peritoneum (15%). This distribution aligns with existing litera-ture, particularly in regions with high tuberculosis prevalence [3,9,24]. The predominance of central nervous system involvement highlights the severity of pediatric extrapulmonary tuberculosis and the importance of early diagnosis. These forms are associated with high morbidity and mortality in the absence of prompt treatment [31].

The case-fatality rate associated with extrapul-monary tuberculosis was 10%. This high rate is largely explained by the frequency of severe forms, particularly central nervous system in-volvement, as well as delays in diagnosis and treatment observed in settings with limited resources [1,4,11,24]. Similar mortality rates have been reported in several African and Asian studies, ranging from 8% to 20%, especially in cases of co-infection with HIV or severe malnutrition [3,28]. Conversely, countries with early diagnosis and adequate diagnostic facilities generally report lower mortality rates, below 5%, highlighting the significant impact of early intervention [23,24].

The clinical course of the different forms of tuberculosis depends on the affected area. Lymph node, pleural, and peritoneal forms responded well to antituberculosis treatment, with gradual resolution of clinical signs during follow-up. Osteoarticular forms required prolonged immobilization and specialized monitoring, in addition to antituberculosis therapy, depending on the specific site of involvement. Pericardial involvement was managed with antituberculosis therapy combined with corticosteroid therapy and showed a favorable clinical course with monitoring. Neuromeningeal forms also benefited from corticosteroid therapy in addition to antituberculosis treatment. These forms were associated with the poorest prognosis, with a 17% mortality rate and neurological sequelae occurring in 26% of survivors. These results align with World Health Organization recommendations that classify neuromeningeal tuberculosis as a severe form requiring specific management, combining appropriate antituberculosis therapy with adjunctive corticosteroid therapy, due to the high risk of neurological complications, sequelae, and mortality [20].

However, our retrospective, single-center approach limits the generalizability of the results to the entire Central African pediatric population. Furthermore, unequal access to sensitive diagnostic tests may have underestimated the actual proportion of extrapulmonary tuberculosis in our cohort. The CHUPB is the main national pediatric referral center, receiving most critically ill children from Bangui. This means the center provides only partial representation of severe cases. Detailed immunovirological data (CD4 count, viral load, and antiretroviral treatment adherence) were unavailable. Similarly, we could not document the causes of antituberculosis treatment failure in children with a history of pulmonary tuberculosis using medical records. This includes treatment adherence, interruptions, and possible drug resistance.

## Conclusion

Extrapulmonary tuberculosis in children at CHUPB remains common and severe, with diagnostic delays, insufficient vaccination cover-age, and high mortality. To save lives and make progress toward Sustainable Development Goal 3, the Central African government must strengthen early tuberculosis screening. It is also necessary to ensure complete BCG vaccination and to improve access to specialized pediatric care as well as to anti-tuberculosis treatments. Finally, raising awareness among families and health care workers remains a crucial component of the fight against this disease. These actions are essential to reduce morbidity and mortality associated with pediatric tuberculosis.

## Funding

No specific funding was received for the conduct of this study. The authors declare that the preparation of this manuscript did not receive any grants or institutional or commercial support.

## Author’s contributions

BOGNING MEJIOZEM Olivier Brice: conceptualization, methodology, data management, drafting, revision and editing, drafting of the original version, study administration. SÉPOU Yanza Marie Christine Awa: supervision, revision, and editing. NGOZIKA Laurice: conceptualization, investigation, data management, study administration. GBELESSO Junior Sylvère: investigation, data management, and writing. NGUERASSEM Abdias: data analysis and proofreading. HENKO KINDING Yannick Wilson: data collection and critical review. GODY Jean Chrysostome: supervision, study validation

## Conflicts of interest

The authors declare no conflicts of interest.
